# Differential gene expression (DGE) analysis in persons with a history of giardiasis

**DOI:** 10.1186/s13568-023-01657-1

**Published:** 2024-01-03

**Authors:** Parnia Saeedi, Gilda Eslami, Masoud Tohidfar, AbbasAli Jafari-Nodushan, Mahmood Vakili

**Affiliations:** 1 Department of Medical Parasitology and Mycology, School of Medicine, Shahid Sadoughi University of Medical Sciences, Yazd, Iran; 2 Department of Cell & Molecular Biology, Faculty of Life Sciences & Biotechnology, Shahid Beheshti University, Tehran, Iran; 3 Department of Community and Preventive Medicine, Health Monitoring Research Center, School of Medicine, Shahid Sadoughi University of Medical Sciences, Yazd, Iran

**Keywords:** *Giardia duodenalis*, Cancer, Candidate genes, Differential gene expression

## Abstract

Giardiasis, which is caused by *Giardia duodenalis*, has clinical symptoms such as steatorrhea and can be very dangerous in children*.* In addition, some documents reported that this parasite is present inside the tissue of patients with cancer. In this study, we analyzed the gene expression profiles of some main genes important to apoptosis and anti-apoptosis in humans.

Expression profile arrays of Genomic Spatial Event (GSE) 113666, GSE113667, and GSE113679 obtained from Gene Expression Omnibus were used for meta-analysis using R commands. Cytoscape and STRING databases used the protein–protein Interaction network. Then, the Kyoto Encyclopedia of Genes and Genomes and Gene Ontology analysis was performed. Similar genes in *Homo sapiens* were identified using Basic Local Alignment Search Tool analysis. The validation was performed on eight people using real-time Polymerase chain reaction. In addition to the candidate genes, the gene expression of some other genes, including *Serine/Threonine Kinase* 1 (*AKT1*), *Cyclin Dependent Kinase Inhibitor 2A* (*CDKN2A*), *Kirsten Rat Sarcoma* (*KRAS*), and *Phosphatidylinositol-4,5-Bisphosphate 3-Kinase Catalytic Subunit Alpha* (*PIK3CA*) were also examined. Analysis of the expression of *serum amyloid A1* (*SAA1*)*, Regenerating Islet-Derived 3 Gamma* (*REG3G*)*,* and *REG3A* genes did not show any difference between the two groups of healthy and diseased people. Examining the mean expression of the four genes *AKT1, CDKN2A, KRAS,* and *PIK3CA* showed that three genes of *AKT1, CDKN2A,* and *KRAS* had increased expression in people with a history of giardiasis compared to healthy people. We showed that the gene expression pattern differs in apoptosis and anti-apoptosis signaling in people with a history of giardiasis. *Giardia duodenalis* seems to induce post-non-infectious symptoms with stimulation of human gene expression.

## Introduction

Giardiasis is one of the most common parasitic infectious diseases of the digestive system worldwide caused by *Giardia* *duodenalis* (Vivancos et al. 2018). *G. duodenalis* is a flagellate unicellular eukaryotic parasite (Barwick et al. 2000) transmitted through contaminated water and food, even though direct transmission has also been reported (Adam 2021). Two forms of *G. duodenalis* are present in the life cycle, including trophozoite and cyst (Bernander et al. 2001). The prevalence of giardiasis in developed countries is 2–7%, and in developing countries, it is 20–30% (Dixon 2011). From 2000 to 2020, giardiasis was reported in around 5.2% of fields (Teimouri et al. 2021). While asymptomatic giardiasis is common, symptomatic infections occur more often in young children than in adults (Allain and Buret 2020), including diarrhea, steatorrhea, malabsorption, anorexia, nausea, weight loss, vomiting, severe abdominal pain, and flatulence (Mørch and Hanevik 2020). Despite significant measures done toward understanding the pathogenesis of giardiasis in the past decades, the pathophysiology of this disease is still under investigation. Epithelial dysfunction during acute and chronic infections has also been reported (Allain & Buret 2020; Teimouri et al. 2021), but the pathophysiology of gastrointestinal manifestations associated with asymptomatic and symptomatic infections remains unclear. *G. duodenalis* is a non-invasive protozoan that infects the small intestine and colonizes the epithelial surface and lumen (Certad et al. 2017). However, there are some documents regarding the presence of *G. duodenalis* trophozoites inside the tissues of the pancreas (Furukawa et al. 2011), stomach (Carroccio et al. 1997), caecum (Carter et al. 2007) or distal small intestine (Halliez and Buret 2013). Correspondingly, some scholars have reported this parasite inside the tissue of cancer patients, such as Terra et al. (Terra et al. 2020) that isolated *G. duodenalis* from the anal mass of a 57-year-old woman with neoplasia; Garg (Garg et al. 2018) who reported the isolation of *G. duodenalis* from the lymph node of a 64-year-old man with cholangiocarcinoma; and Mitchell et al. (Mitchell et al. 2011) isolating the parasite from pancreatic cancer mass. Giardiasis has been recognized as a risk for the development of post-infectious functional gastrointestinal disorders (Dormond et al. 2016; Hanevik et al. 2009; Litleskare et al. 2018; Nakao et al. 2017).

To better understand the complex system of molecular processes and identify pathways and mechanisms involved in cell response to diseases, it is indispensable to use statistical and computational approaches. High-throughput technologies such as microarray, which are being used for gene expression analyses, have made it feasible to study a large number of genes simultaneously in different conditions. This study’s microarray and sample annotation data have been deposited in the NCBI Gene Expression Omnibus (GEO) repository under accession number SuperSeries.

In the present study, we analyzed the expression of some candidate genes in apoptosis signaling in persons with a history of giardiasis compared to healthy people with SYBR Green Real-Time PCR using delta Ct to perform relative quantification of gene expression. First, the Gene Expression Omnibus (GEO) datasets were analyzed to select the candidate genes. This dataset in NCBI has comparable samples processed using the same Platform and includes the experimental variables. Then, more analysis was performed to find the upregulated genes after being infected by *G. duodenalis*. For an accurate evaluation, a meta-analysis was carried out. Meta-analysis is a standard statistical procedure for combining datasets from multiple studies to systematically assess previously published data to derive more comprehensive conclusions about that research field. This technique provides a broad perspective on specific biological questions and more reliable results than individual studies.

Then, the important genes related to the liver that had overexpression after giardiasis infection were selected to evaluate in an experimental study with two groups of healthy people to compare the gene expression of selected genes from datasets in individuals with no history of giardiasis and digestive disorders with the ones in people with the history of giardiasis in the past with no specific symptoms of giardiasis.

## Materials and methods

### Microarray databases

We obtained microarray data from the Gene Expression Omnibus (GEO) database (http://www.ncbi.nlm.nih.gov/geo). Since there were no datasets, including the gene expression profile of the liver from *Homo sapiens* infected with *G. duodenalis*, we used the gene expression profile in the liver from *Mus musculus* after the *Giardia* infection. We received three datasets with accession numbers GSE113679, GSE113666, and GSE113667 regarding *Mus musculus*, which had 32 samples in total, involving 16 samples as control and 16 samples as treatment; the GSE113697 series on the GPL24228 platform, the GSE113666 series on the GPL10333 platform, and the GSE113667 series on the GPL19795 platform.

### Meta-analysis of the gene expression in datasets

We checked the quality control of each data set using boxplot diagrams and density histograms. We also removed the noises in the meta-analysis of microarray data to use datasets with different platforms by effect Batch method and by ComBat and SVA packages in R software (version 4.2.1). The comparison of genes was carried out with other expression genes (DEGs) using the limma package in R software. The DEGs were selected with commonly used equal change ∣log2 (FC)∣ > 1 threshold and *p*-value < 0.05.

### Functional analysis

We performed GO, KEGG pathway, and REACTOME pathway using the Database for Annotation, Visualization, and Integrated Discovery (DAVID; https://david.ncifcrf.gov/) https://david.ncifcrf.gov/for DEGs. The significance criterion was set for screening enriched GO terms, KEGG, and REACTOME pathways at *p* < 0.05.

### PPI network construction and hub gene selection

We predicted the PPI network of target genes using an online STRING database (http://string-db.org) and subsequently visualized it in Cytoscape (version 3.9.0). Then, we used the cytoHubba plug-in in Cytoscape and identified ten hub genes.

The three genes with significant expression, including *Saa3, Reg3g,* and R*eg3b,* were detected in *Mus musculus*. Subsequently, due to our goal to assess the most important hub genes in *Homo sapiens*, we detected the *Homo sapiens* genes, which were equivalent to *Saa3, Reg3g,* and R*eg3b* in *Mus musculus*. The mentioned genes in humans were *SAA1, REG3G, and REG3A*. Additionally, the expression of the above genes was analyzed using SYBR Green real-time PCR.

### AKT1, KRAS, PIK3CA, and CDKN2A expression profile

According to microarray studies, our samples did not express three genes, *SAA1*, *REG3A*, and *REG3G*. Consequently, we added and assessed *AKT1*, *KRAS*, *PIK3CA*, and *CDKN2A* genes implicated in cancer development pathways by referring to various articles. The *AKT* gene inhibits cell apoptosis and stimulates cell proliferation following activation of protein kinase B, serine/threonine kinase (Abeyrathna and Su 2015). The *PIK3CA* gene encodes the catalytic subunit of phosphatidylinositol 3-kinase (PI3K) and is one of the most common genes in tumor malignancies (Cai et al. 2020). *KRAS* gene, a member of the RAS superfamily, encodes a small GTPase that regulates diverse cellular processes, including cell proliferation, differentiation, survival, and migration (Downward 2003). *CDKN2A* is an important tumor suppressor gene (Foulkes et al. 1997).

### Primer design

Finally, we designed specific primer pairs for key genes using Primer3 Software (Table 1).

**Table 1:** The sequences of the used primers in this study

Primer name	Sequence (5′-3′)	Target gene
REG3A-F	GCCCTCTGGAAACCTGGTGTCTGT	*REG3A*
REG3A-R	TGCTACTCCACTCCCAACCTTCTCCA	
REG3G-F	GCCCTCTGGAAAACTGGTGTCTGT	*REG3G*
REG3G-R	TGCTACTCCACTCCCATCCATCTCCA	
SAA1-F	CGGGGGAACTATGATGCTGCCAAA	*SAA1*
SAA1-R	AGCAGGTCGGAAGTGATTGGGGTCT	
AKT1-F	ACCCTTCAAGCCCCAGGTCA	*AKT1*
AKT1-R	CGCTCGCTGTCCACACACTC	
KRAS-F	GGGAGGGACTAGGGCAGTTTGGA	*KRAS*
KRAS-R	CTTGGCACACCACCACCCCAAAATC	
PIK3CA-F	AGCCCCGAGCGTTTCTGCTTTGG	*PIK3CA*
PIK3CA-R	TGCCCCACAGTTCACCTGATGATGG	
CDKN2A-F	GGGGCACCAGAGGCAGTAACC	*CDKN2A*
CDKN2A-R	ACGAAAGCGGGGTGGGTTGT	
GAPDH-F	TTGACGCTGGGGCTGGCATT	GAPDH
GAPDH-R	ATGAGGTCCACCACCCTGTTGCTGT	

### Patients

In this study, two groups with a history of giardiasis and one without parasite exposure were examined (Table 2). Individuals with a history of giardiasis were coded as A22, A4, and A8. Only those patients with the code of A2 had digestive symptoms. The healthy people with no history of giardiasis were coded as A1, A3, A5, A6, and A7. None of them had digestive symptoms, but sample A7 has rheumatism.

**Table 2:** Grouping of studied people

Sample number	Age	Sexuality	Duration infection	Disease to gene analysis	Symptoms
A1	26	Female	–	–	–
A2	34	Female	1 month	10 months	Diarrhea, excessive stress
A3	33	Female	–	–	–
A4	22	Male	2 weeks	5 months	–
A5	23	Male	–	–	–
A6	27	Female	–	–	–
A7	23	Female	–	–	Rheumatism
A8	21	Male	1 month	15 years	–

### Sampling

In this study, blood sampling was done from eight people, including five healthy ones and three with giardiasis who had a history of giardiasis for three months to 15 years before selection. Blood collection was done and immediately used for RNA extraction.

### RNA extraction and cDNA synthesis

The total RNA was extracted from each blood sample using a total RNA extraction kit (Parstous, Mashhad, Iran) according to the manufacturer’s instructions. We used DNase I (CinnaGen, Tehran, Iran) to treat the extracted RNA to avoid genomic contamination. The RNA quantity was done using Nanodrop (Thermo Fisher Scientific, USA). The complementary DNA (cDNA) was synthesized from total RNA (1 μg) using the RevertAid First-Strand cDNA Synthesis Kit (Thermo Fisher Scientific, USA) based on the manufacturer’s instructions.

### Gene expression

We assessed the gene expression of the genes (Table 3) using SYBR Green Real-Time PCR. The housekeeping gene of GAPDH was considered an internal or endogenous control for normalization purposes. Amplification was done in a total volume of 10 μl containing 1 μl of cDNA, 5 μl of SYBR Green Real-Time Master Mix (2x), and 900 nM of each primer using a StepOne thermocycler (ABI, USA). The thermal condition of each reaction was 95 °C for 10 min to the first denaturation, followed by 40 cycles of 95 °C for 15 s and 60 °C for 1 min. Then, the melt curve analysis was set up for each run. All tests were done in duplicate. We determined the relative amount of amplification by each primer pair based on the threshold cycle value (Ct) of the gene of interest, normalized to the reference gene GAPDH. The gene expression analysis was performed using delta delta Ct.

**Table 3:** The genes characterizes and their roles assessed in this study

Gene name	Description	Specific biological role
*SAA1*	Serum amyloid A1	Major acute phase protein
*REG3A*	Regenerating family member 3 alpha	Tumor suppressor
*REG3G*	Regenerating family member 3 gamma	Antimicrobial peptides
*AKT1*	Serine/threonine kinase 1	Regulate metabolism, proliferation, cell survival, growth and angiogenesis
*KRAS*	Kirsten rat sarcoma viral oncogene homolog	Regulation of cell division
*PIK3CA*	Phosphatidylinositol-4,5-bisphosphate 3-kinase catalytic subunit alpha	Participates in cellular signaling in response to various growth factors
*CDKN2A*	Cyclin-dependent kinase inhibitor 2A	Tumor suppressor

### Statistical analysis

The collected data were computerized with SPSS Software version 18, and the results were presented as tables, graphs, and statistical indices. The normality of the data was quantified by the Kolmogorov*–*Smirnov statistic. T-test was used for comparison of the gene epression. The *p*-value less than 0.05 was considered significant.

## Results

### Meta-analysis of microarray data

Following the data quality control assessment, the data with a low-quality sample were excluded from further examination. The results of data analysis showed that DEGs contained 34,847 common genes, including 27 genes with increased expression or up (FDR ≤ 0.05, Log FC ≥ 0.7) and 18 genes with decreased expression or down (FDR ≤ 0.05, Log FC ≤ -0.7).

### PPI network construction and hub gene selection

The Cytoscape database and STRING analysis used to construct a Protein–Protein Interaction network showed that the PPI network consisted of 38 nodes and 10 edges. The PPI network revealed that Reg3g and Reg3b genes had the most interactions. Then, we identified the top 10 hub genes in Cytoscape using cytoHubba.

### Functional analysis

**Fig. 1:**
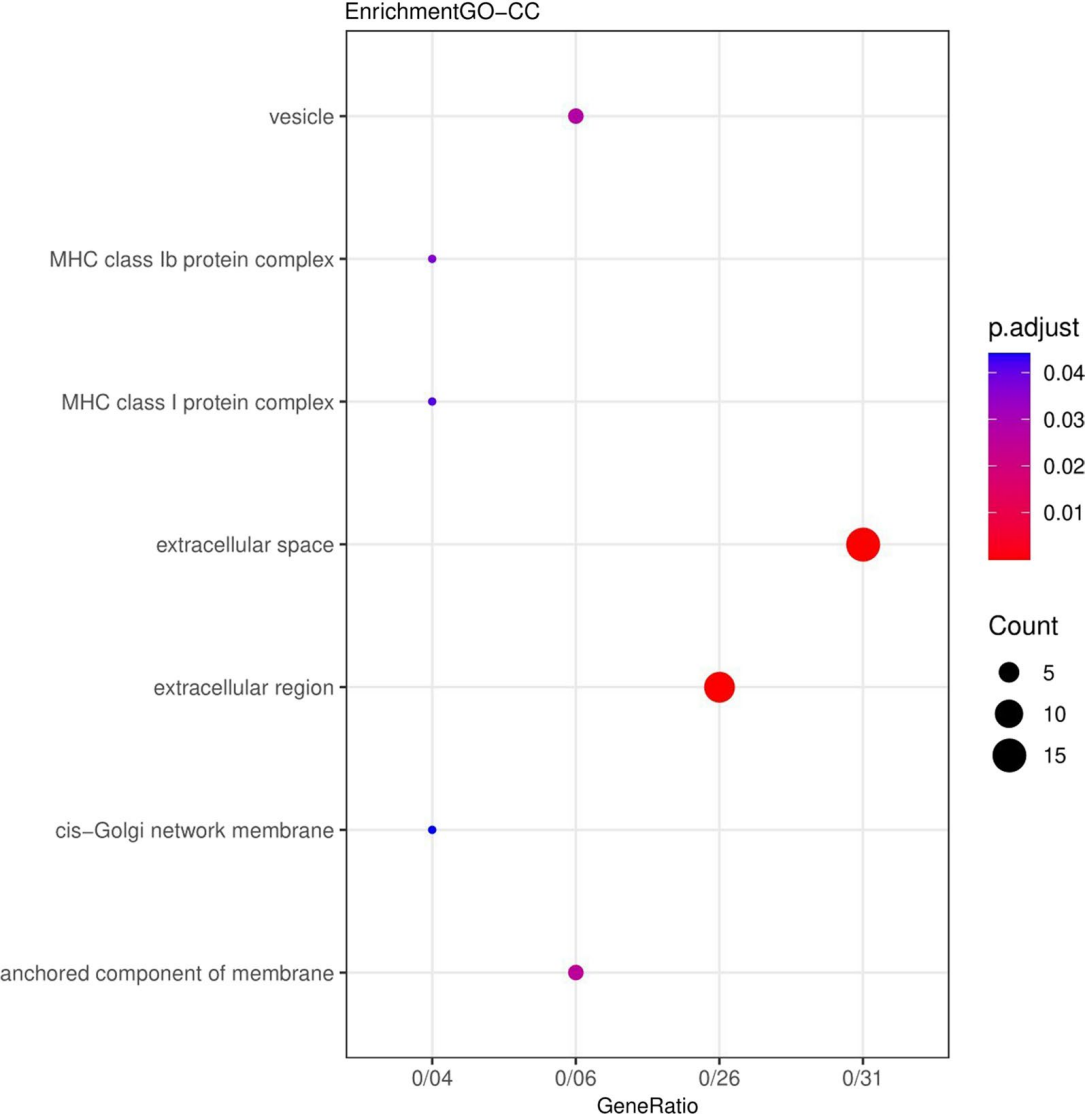
Genes enriched in the range of cellular compartments. Count: indicates the number of genes in each path, the larger the circle, the more genes. p. adjusted: the same p. Value that includes FDRs smaller than 0.05. GeneRatio: It is the ratio of the number of genes of each GO to the total number of DEGs

**Fig. 2:**
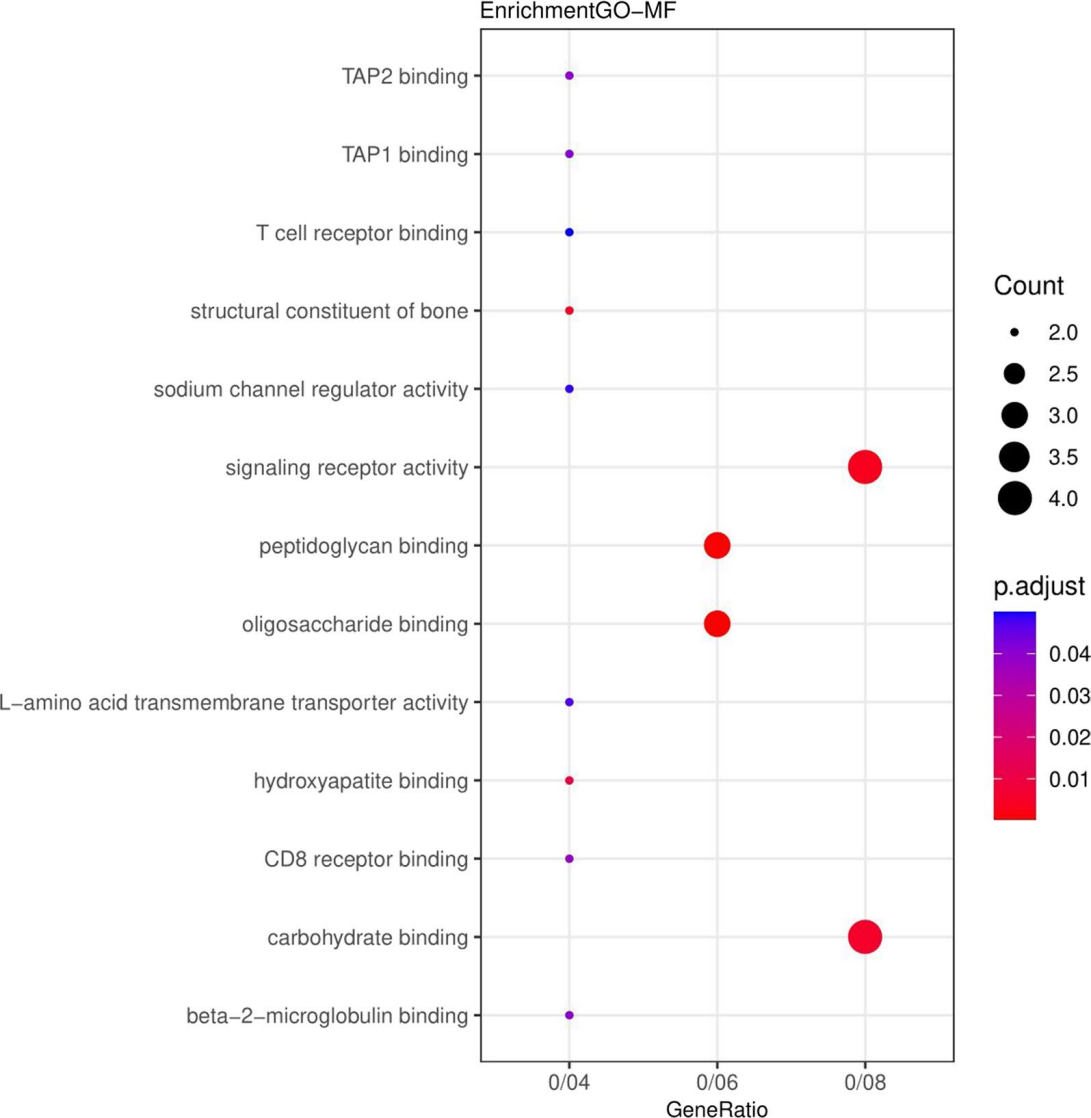
Enriched genes in the range of molecular functions

**Fig. 3:**
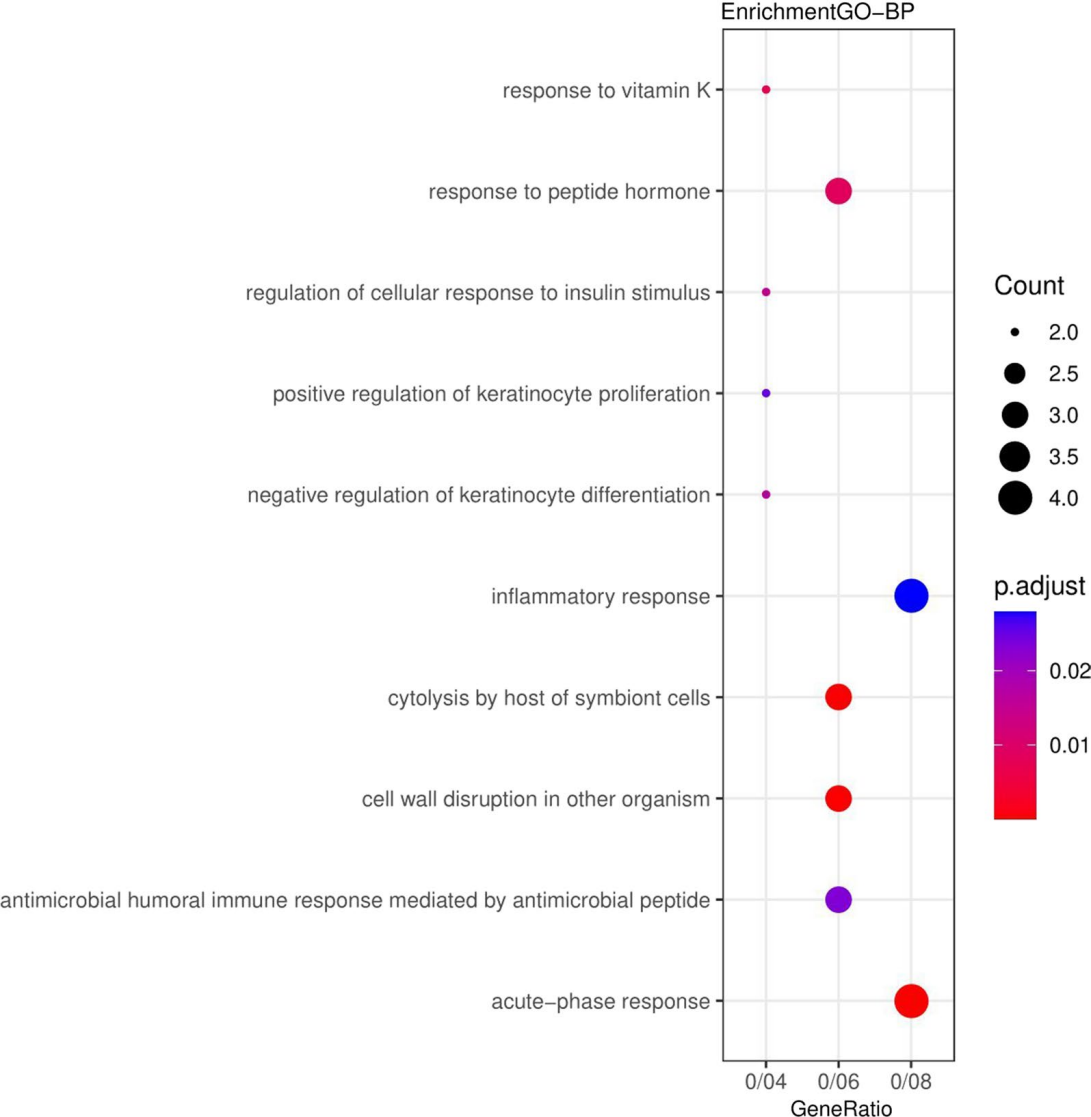
Genes enriched in the range of cellular processes

**Fig. 4:**
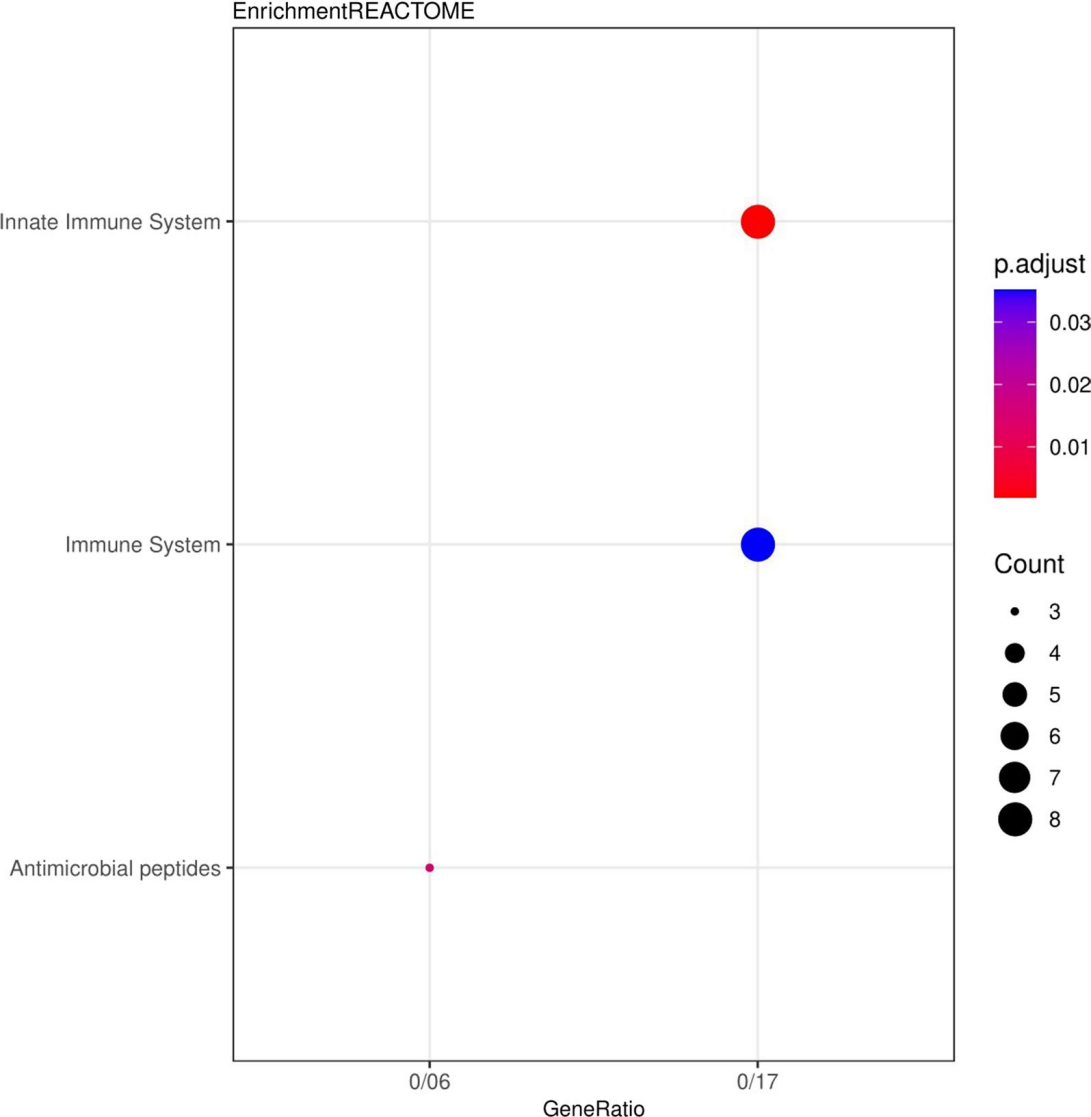
REACTOME enrichment pathway

The results related to the functional role of up and down genes (DEGs) displayed that these genes were significantly enriched in the extracellular space and extracellular region (Fig. 1). Also, for the Molecular Functions category, the genes were enriched in oligosaccharide binding (Fig. 2). In the category of Biological Processes, the genes were enriched in the acute-phase response (Fig. 3). According to KEGG pathway enrichment analysis, the DEGs were significantly enriched in Phagosome pathway, and in REACTOME pathway analysis, the DEGs were significantly enriched in Innate Immune System pathway (Fig. 4).

Consequently, among the genes with increased expression, three genes of *Saa3*, *Reg3b,* and *Reg3g* had the highest expression compared to others, which we chose as key genes. Since our samples were human, we examined three genes, *SAA1*, *REG3G*, and *REG3A*, which correspond to human genes as they do in mice.

### qPCR analysis

**Fig. 5:**
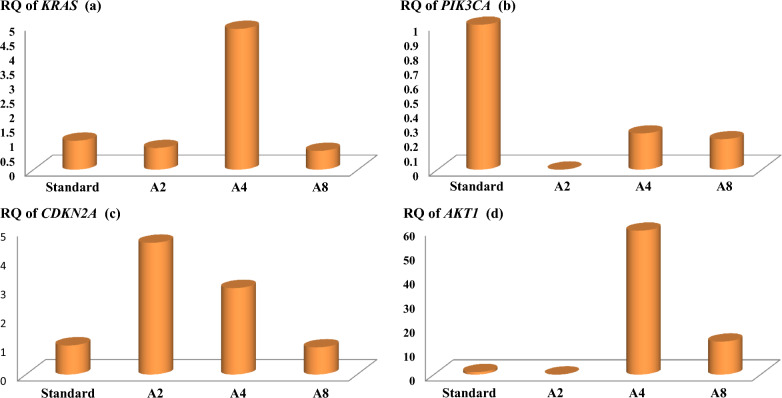
Relative gene expression (RQ) of **a** *KRAS*, **b** *PIK3CA*, **c** *AKT1*, **d** *CDKN2A* in people with giardiasis by Real-time PCR method. Standard: Non-infected group. A2, A4, A8: Sample number of people with giardiasis. **a** The expression of gene *KRAS* was observed to decrease in samples A2 and A8 and increase in sample A4. **b** The expression of gene *PIK3CA* was decreased in samples A4 and A8, and there was no expression in sample A2. **c** The expression of gene *AKT1* was increased in samples A4 and A8, and it was absent in sample A2. **d** The expression of gene *CDKN2A* in samples A2 and A4 had increased expression, and in sample A8, gene expression was almost equal to the standard sample

The relative expression level of the *AKT1* gene in samples with a history of giardiasis disease A4 and A8 increased by 59.63 and 13.57 times, respectively, compared to the average expression of the *AKT1* gene in healthy subjects. No expression was observed in sample A2 (Fig. 5;* p* > 0.05). The relative expression of the *CDKN2A* gene in samples with a history of giardiasis A2 and A4 increased by 4.56 and 2.99 times, respectively, compared to the average expression of the *CDKN2A* gene in healthy individuals, and the expression of the gene in sample A8 was almost equal to the standard (*p* > 0.05). The relative expression of the *KRAS* gene in samples with a history of giardiasis A2 and A8 decreased by 0.75 and 0.65 times, respectively, compared to the average expression of the said gene in healthy individuals. Gene expression in sample A4 was 4.86 compared to the average expression of the said gene in healthy people, showing an increase (*p* > 0.05). The relative expression level of the *PIK3CA* gene in samples with a history of giardiasis disease A4 and A8 decreased by 0.25 and 0.21 times, respectively, compared to the average expression of the said gene in healthy individuals, and no expression was observed in sample A2 (*p* > 0.05). The fold changes of the *AKT1*, *KRAS*, *CDKN2A*, and *PIK3CA* genes in people with giardiasis are shown in Fig. 5.

## Discussion

Based on knowledge, a *Giardia* infection may result in cancers of the colorectal area and liver (Mahdavi et al. 2022). Also, there are some case reports regarding associations of *Giardia* with pancreatic cancer (Hurník et al. 2019) and gall bladder cancer (Nagasaki et al. 2011). Therefore, it is necessary to investigate the possible long-term effects of this parasite on other areas of the digestive tract, especially in the liver, pancreas, gall bladder, etc. In this study, we evaluated the persons with a history of giardiasis. These people, like non-infected people, were both females and males, and the age range of both groups was almost equal, from 21 to 33. The non-infected group, unlike the infected group, has no symptoms. In the infected group, only sample A2 showed digestive symptoms, including diarrhea and neurological symptoms such as excessive stress.

In the first step, the expression pattern of liver genes of mice infected with giardiasis was compared to healthy mice using bioinformatics methods to select the candidate genes. Then, to verify the gene expression pattern in people infected with parasites, it was compared with healthy people using laboratory methods. This study’s bioinformatics section found that three genes, Saa3, Reg3b, and Reg3g, had the highest expression in mice, similar to *SAA1, REG3A,* and *REG3G* genes in humans. We showed that none of these genes were expressed in the studied samples. It may be due to some different pathways in mice and humans. Mice strains are often used to model human disease states, therapeutic principles, and drug efficacy testing. However, a direct translation of mouse experimental data to human pathological events often fails due to substantial differences in the immune systems of both species (Zschaler et al. 2014). Since it is quite obvious that humans and mice differ in size, behavior, lifespan, living conditions, ecological situation, and other characteristics (Russell 1985), modern genetic approaches allow detailed analysis of genomes in humans and mice. Due to the common progenitor of both species, significant amounts of orthologous genes are found along with a sufficient number of individual genes associated with the species (Waterston et al. 2002). There are 15.187 genes from both species that are functionally associated, which means 75% of mouse genes and 80% of human genes are orthologous. These one-to-one orthologs have nucleotide and amino acid identities of 85.3% and 88.2%, respectively (Church et al. 2009). In defense against invading microorganisms, resistance mechanisms dominate humans, while tolerance mechanisms dominate mice (Schneider & Ayres 2008). Species differences exist in the activation and function of effector molecules released by immune cells to control pathogens (Warren et al. 2010). In addition, several pathogens found predominantly in humans but rarely or not at all in mice include *Mycobacterium tuberculosis*, *Mycobacterium leprae*, *Shigella flexneri*, *Plasmodium falciparum*, and viruses such as measles and dengue virus (Murray and Wynn 2011). Since the main immune mechanisms in mice are entirely different from those in humans, and the results of the present study also confirmed this, it seems that generalizing the results of experiments in mice to humans is not a suitable method.

The present study found that the mean relative expression level of the *AKT* gene in people with a history of giardiasis is increased compared to healthy people. However, among this study’s samples, one sample had lower AKT gene expression related to a woman. *AKT* signaling pathway is involved in inhibiting cell apoptosis and stimulating cell proliferation following activation of protein kinase B and serine/threonine kinase (Abeyrathna and Su 2015). Based on these studies, it has been determined that *Giardia* plays a role in triggering the apoptotic pathway in humans. Liu et al. (2020) showed that *G. duodenalis* induces Caco-2 cell apoptosis through a caspase-dependent pathway mediated by ROS and mitochondria (Liu et al. 2020). Therefore, it may be a natural reaction of the immune cell against the apoptosis effect of *Giardia* at the site of the disease; however, it is suggested that the AKT expression be investigated in patients with acute symptoms. Yang et al. (2022) also observed that *AKT* overexpression in p38/ERK/AKT/NF-kB signaling could inhibit anti-intestinal epithelial cell (IEC) apoptosis during giardiasis mediated by COX-2-mediated and ROS/NO production (Yang et al. 2022). The high *AKT* gene expression is a way to control the destruction caused by this parasite. Our results indicated that A2 showed low expression of the *AKT* gene. Zhao (2021) observed that *G. duodenalis* extracellular vesicles (GEV) could increase the levels of parasite-induced inflammatory response in mouse macrophages through activation of *p38*, *ERK*, and *NF-kB* signaling pathways, and activation reduces the *AKT* signaling pathway (Zhao et al. 2021). Li et al. (2017) presented that macrophages play a role in the immune response to giardiasis by activating AKT/MAPK signaling (Li et al. 2017). A document suggests exposure of the human’s biofilms to the gut microbiota of *Giardia* spp. induces the release of buoyant planktonic bacteria, leading to the induction of epithelial apoptosis, promoting bacterial transmigration, and a pro-inflammatory reaction. It suggests that it may increase the production of CXCL8 (interleukin-8) (Beatty et al. 2017). On the other hand, the *PI3K/AKT* signaling pathway is one of the major pathways that regulates IL-8 expression and enhances tumor cell migration or invasion. However, in our study, *PI3K* had reduced expression in all individuals with a history of giardiasis. The *PIK3CA* gene encodes the catalytic subunit of phosphatidylinositol 3-kinase (PI3K), one of the most common genes in tumor malignancies. Activation of the *PI3K* pathway leads to the induction of the cyclooxygenase-2 (COX-2) enzyme and the production of immunosuppressive prostaglandin E2 (PGE2) (Cai et al. 2020). *PIK3CA* gene is confirmed in various cancers, including colorectal, breast, head, and neck (Cai et al. 2020), ovarian cancer (Shayesteh et al. 1999), and cervical cancers (Ma et al. 2000). Since this gene is the primary gene in upstream of the *AKT* gene, it seems that some other mechanisms are involved in the over-expression of *AKT* in two samples of A4 and A8.

The mean of *KRAS* gene expression in people with a history of giardiasis was higher than in healthy people, although the high expression was reported just in the A4 sample. The *KRAS* gene, a member of the *RAS* superfamily, encodes a small GTPase that regulates diverse cellular processes, including cell proliferation, differentiation, survival, and migration (Downward 2003). *KRAS* gene is involved in several cancers, namely lung cancer, colorectal cancer (Wang and Fakih 2021), pancreatic cancer (Grant et al. 2016), and ovarian cancer (Jumaa 2022). As with AKT, the high expression of the KRAS gene may be related to the prevention of apoptosis by *Giardia*. On the other hand, two samples with a history of giardiasis showed low expression of *KRAS.*

In this study, we demonstrated that the mean relative expression of the *CDKN2A* gene in people with a history of giardiasis is more than the expression of this gene in healthy people. However, it should be noted that sample A8 displayed low expression of this gene. *CDKN2A* is an important tumor suppressor gene (Foulkes et al. 1997). The *CDKN2A* is a potent cyclin-dependent kinase inhibitor and a critical G1-specific negative regulator that arrests cell cycle progression at the G1-S phase boundary. Loss of its function can lead to uncontrolled cell proliferation (Serrano et al. 1993). *Giardia* may stimulate the *CDKN2A* gene expression for apoptosis (Cánepa et al. 2007). Since sample A8 had a history of giardiasis for more than 10 years, the gene may reduce gene expression after these years.

In conclusion, Genes *AKT1*, *CDKN2A*, *KRAS*, and *PIK3CA* were expressed in our samples (human). We showed that the gene expression of some main genes in apoptosis and anti-apoptosis signaling in people with a history of giardiasis differs. *Giardia duodenalis* seems to induce post-non-infectious symptoms with stimulation of human gene expression. Gene expression patterns can be used as a biomarker. Due to the few samples in this study, we suggest evaluating the studied gene expression in more cases.

## Data Availability

The datasets used and analyzed during the current study are available from the corresponding author upon reasonable request.

## Abbreviations

GEO: Gene expression omnibus
DEGs: Differential expression genes
PPI: Protein–protein interaction
KEGG: Kyoto encyclopedia of genes and genomes
GO: Gene ontology
